# SLC39A5 dysfunction impairs extracellular matrix synthesis in high myopia pathogenesis

**DOI:** 10.1111/jcmm.16803

**Published:** 2021-07-24

**Authors:** Shanshan Dong, Qi Tian, Tengfei Zhu, Kangli Wang, Ganting Lei, Yanling Liu, Haofeng Xiong, Lu Shen, Meng Wang, Rongjuan Zhao, Huidan Wu, Bin Li, Qiumeng Zhang, Yujun Yao, Hui Guo, Kun Xia, Lu Xia, Zhengmao Hu

**Affiliations:** ^1^ Center for Medical Genetics & Hunan Key Laboratory of Medical Genetics School of Life Sciences Central South University Changsha Hunan China; ^2^ Department of Critical Care Medicine Shenzhen Third People's Hospital Shenzhen Guangdong China; ^3^ National Clinical Research Center for Geriatric Disorders Department of Geriatrics Xiangya Hospital Central South University Changsha Hunan China; ^4^ Hunan Key Laboratory of Molecular Precisional Medicine Central South University Changsha Hunan China; ^5^ Hunan Key Laboratory of Animal Models for Human Diseases Central South University Changsha Hunan China

**Keywords:** ECM, high myopia, SLC39A5, Smad, TGF‐β signalling, zinc

## Abstract

High myopia is one of the leading causes of visual impairment worldwide with high heritability. We have previously identified the genetic contribution of *SLC39A5* to nonsyndromic high myopia and demonstrated that disease‐related mutations of *SLC39A5* dysregulate the TGF‐β pathway. In this study, the mechanisms underlying SLC39A5 involvement in the pathogenesis of high myopia are determined. We observed the morphogenesis and migration abnormalities of the SLC39A5 knockout (KO) human embryonic kidney cells (HEK293) and found a significant injury of ECM constituents. RNA‐seq and qRT‐PCR revealed the transcription decrease in *COL1A1*, *COL2A1*, *COL4A1*, *FN1* and *LAMA1* in the KO cells. Further, we demonstrated that TGF‐β signalling, the regulator of ECM, was inhibited in SLC39A5 depletion situation, wherein the activation of receptor Smads (R‐Smads) via phosphorylation was greatly blocked. SLC39A5 re‐expression reversed the phenotype of TGF‐β signalling and ECM synthesis in the KO cells. The fact that TGF‐β signalling was zinc‐regulated and that SLC39A5 was identified as a zinc transporter urged us to check the involvement of intracellular zinc in TGF‐β signalling impairment. Finally, we determined that insufficient zinc chelation destabilized Smad proteins, which naturally inhibited TGF‐β signalling. Overall, the SLC39A5 depletion–induced zinc deficiency destabilized Smad proteins, which inhibited the TGF‐β signalling and downstream ECM synthesis, thus contributing to the pathogenesis of high myopia. This discovery provides a deep insight into myopic development.

## 1 INTRODUCTION

Myopia is a condition of refractive error causing light to focus in front of the retina, with axial elongation as its most common pathology. High myopia, clinically defined as a refractive error of at least −6.00 diopters (D) or an axial length >26 mm,^1^ is one of the leading causes of visual impairment worldwide^2^, ^3^ with a significantly increased prevalence.^3^, ^4^ Thus, the prevention of high myopia has become a global public health problem. Pedigree analyses and twin studies identified high myopia highly heritable.^5^, ^6^ Even though numerous loci (1q41, 4q25, 8p23, etc.) and causative genes (*ZNF644*, *CCDC111*, *SCO2*, etc.) have been identified,^7^ the pathogenic mechanism of high myopia remains unclear.

Recently, scleral extracellular matrix (ECM) synthesis or remodelling has been proposed as an underlying cause of high myopia.^8^, ^9^, ^10^ According to this hypothesis, scleral collagen synthesis is regulated during myopia progression,^9^ while scleral ECM is remodelled in myopic animal.^10^ ECM synthesis and remodelling are targets of the TGF‐β signalling pathway, one of the most reproducibly dysregulated pathways in myopia development.^10^, ^11^, ^12^, ^13^ Total or isoform‐specific scleral TGF‐β is frequently altered during myopia progression.^9^, ^10^, ^14^

In the previous study, we first identified *SLC39A5* as a high myopia‐associated gene, and mutations of *SLC39A5* dysregulated the BMP/TGF‐β pathway.^15^ Further research confirmed *SLC39A5* as one of the top three genes contributing to nonsyndromic high myopia.^16^ This study aimed to investigate the mechanisms underlying SLC39A5 involvement in the pathogenesis of high myopia. We found that SLC39A5 played a key role in maintaining intracellular zinc homeostasis. SLC39A5 depletion–induced zinc deficiency directly destabilized Smad proteins, and Smads instability further impaired TGF‐β signalling–mediated ECM synthesis, thus contributing to the pathogenesis of high myopia.

## 2 MATERIALS AND METHODS

### 2.1 Cell culture

HEK293 cells and HEK293T cells were cultured in Dulbecco's modified Eagle's medium (DMEM) (Gibco Cat# C11995500BT) containing 10% foetal bovine serum (FBS) (Gibco Cat# 10099‐141). Human lymphocyte cells (M16345, M16346, M16349, M16344, M19118 and M21932) from a high myopia pedigree carrying SLC39A5 mutations reported previously^15^ were cultured in Roswell Park Memorial Institute (RPMI) (Gibco Cat# C11875500BT) containing 20% FBS. All cells were maintained at 37°C with 5% CO_2_ in a humidified incubator and passaged every 2–3 days. All members were recruited for blood collection after providing informed consent. The study was approved by the Institutional Review Board of the Center for Medical Genetics & Hunan Key Laboratory of Medical Genetics and adhered to the tenets of the Declaration of Helsinki.

### 2.2 SLC39A5 knockout cell line construction

According to the protocols from Zhang laboratory (http://www.genome‐engineering.org/gecko/), target‐specific sgRNA was cloned into the lentiCRISPRv2 vector (sgRNAs are listed in Table S1). Then, lentiviruses were packaged in HEK293T cells by co‐transfection of CRISPR‐sgRNA/pVSVG/pspAX2. The supernatants containing viral particles were collected 48 h after transfection and filtered (0.22 μm pore size). Then, HEK293 cells were transduced with viral supernatant supplemented and selected with 1 μg/ml puromycin. After transfection, cells were detached and seeded in 10 ml Petri dishes at a density of 200 cells per plate. Puromycin (2 µg/ml) was added to allow the selection of positive clones during the following two weeks. Positive clones were isolated and transferred to six‐well plates. The SLC39A5 knockout clones were validated by direct Sanger sequencing (primers are listed in Table S2). The negative control cell line was generated from HEK293 transformed with the identical lentiviral vector lacking specific sgRNAs.

### 2.3 Zinc quantification

Zinc quantification assay (Abcam Cat# ab102507) was performed for accurate measurement of zinc levels according to the manufacturer's instructions. In brief, cells were harvested with NP‐40 lysis buffer (EDTA‐free), and proteins were deproteinized by an equal volume of 7% TCA Solution Buffer. The supernatants were transferred to new clean tubes after centrifugation. 200 μl Zinc Reaction Buffer was applied to 50 μl tested samples in a 96‐well plate. After a 10‐min incubation at room temperature, the OD_560_ was measured in a microplate reader. The zinc standard curve was plotted after subtracting the background value, and the tested sample concentration was calculated from the standard curve.

### 2.4 RNA‐seq and analysis

Total RNA for RNA‐seq library was extracted using TRIzol (Invitrogen Cat# AM9738), validated on a NanoDrop 1000 Spectrophotometer (Thermo) and quantitated using Qubit (Thermo). RNA‐seq libraries were prepared using the TruSeq RNA Sample Prep Kit v2 (Illumina Cat# RS‐122‐2001) according to the standard Illumina library preparation procedure. In brief, purified RNA was poly‐A–selected and fragmented, followed by first and second strand cDNA synthesis. Double‐stranded cDNA was processed from end repair to PCR amplification according to the library construction steps. Libraries were purified using AMPure XP beads (Beckman Coulter Cat# A63882), then validated for the appropriate size on an Agilent 2100 Bioanalyzer (Agilent) and quantitated using Quantitative Real‐Time PCR (qRT‐PCR) (TaqMan Probe). Library pools were clustered and run on an Illumina HiSeq 4000 platform (Illumina), according to the manufacturer's recommended protocol. The sequencing data were filtered with SOAPnuke (v1.5.2), then mapped to the reference genome using HISAT2 (v2.0.4) and aligned to the reference coding gene set using Bowtie2 (v2.2.5). Differential gene expression analysis between the target and reference sets of treatments was determined using DESeq2 (https://bioconductor.org/packages/release/bioc/html/DESeq.html). Enrichment analysis of annotated different expressed genes was performed with the Phyper (https://en.wikipedia.org/wiki/Hypergeometric_distribution) based on the hypergeometric test.

### 2.5 qRT‐PCR validation

Total RNA was extracted by TRIzol (Invitrogen Cat# 15596018) according to the manufacturer's instructions. The cDNA was obtained by reverse transcription‐PCR using RevertAid First Strand cDNA Synthesis Kit (Thermo Cat# K1622). qRT‐PCR was conducted with the Maxima SYBR Green qPCR Master Mix (Thermo Cat# K0251) (primers are listed in Table S3). All samples were run in triplicate. qRT‐PCR data were analysed by LightCycler 96 software. Relative candidate gene mRNA levels were normalized to those of β‐actin or GAPDH. A value of *p *< 0.05 was considered to be statistically significant.

### 2.6 Plasmid construction and transient transfection

The Smad1 zinc‐binding site substitutions were generated to assess the correlation between zinc‐binding capacity and protein stability. Smad1 WT plasmid was brought from OriGene (OriGene Cat# RC200299), and the four zinc‐binding site substitutions (C64A, C109A, C121A and H126A) were separately generated using Fast multiSite Mutagenesis System (Transgen Biotech Cat# FM201‐01) (primers are listed in Table S4).

SLC39A5 rescue plasmids were projected to restore the SLC39A5 expression of the KO cells. The pcDNA3.1‐SLC39A5‐Flag plasmid used previously^15^ was introduced with an NGG synonymous mutation in the PAM sequence using Fast multiSite Mutagenesis System (Transgen Biotech Cat# FM201‐01) (primers are listed in Table S5). Finally, the rescue sequence was cloned into the pLVX‐IRES‐puro vector to construct the KO rescue plasmid.

*SLC30A1* shRNA interference plasmids were also constructed to rescue the poor intracellular zinc level of SLC39A5 KO cells by suppressing the zinc efflux baseline. The three targeting sequences were obtained from the siRNA sequence database (Thermo) and then generated into the pFUGW‐lentiviral vector between the XbaI and BamHI restriction sites, respectively (primers are listed in Table S6).

Transient transfections were performed with Lipofectamine™ 2000 (Invitrogen Cat# 11668019) according to the manufacturer's instructions.

### 2.7 Immunoblotting

Cells were lysed by Nonidet P‐40 (NP‐40) buffer (Beyotime Biotechnology Cat# P0013F) for collagen immunoblot or by SDS lysis buffer for other immunoblots. Protein concentration was determined by BCA Protein Assay Kit (Thermo Cat# 23227). Collagens were separated by Precast‐GLgel Hepes Native‐PAGE gel (Biotech Cat# C601100) electrophoresis, and the others were separated by SDS‐polyacrylamide gel electrophoresis, then transferred to the polyvinylidene fluoride (PVDF) membranes and blocked in 5% nonfat milk (5% BSA for phospho‐antibody) in 1% PBST (phosphate‐buffered saline and 1% Triton). After incubated with primary antibodies (Collagen Ⅰ: Abcam Cat# ab34710; Collagen Ⅱ: Abcam Cat# ab34712; Collagen Ⅳ: Abcam Cat# ab6586; fibronectin: Abcam Cat# ab2413; laminin: Abcam Cat# ab7463; Smad1: Cell Signaling Technology Cat# 6944S; Smad2/3: Cell Signaling Technology Cat# 8685S; Smad4: Cell Signaling Technology Cat# 38454S; pSmad1/5/9: Cell Signaling Technology Cat# 9511S; and pSmad2/3: Cell Signaling Technology Cat# 8828S) at recommended dilutions overnight in 4°C, the membranes were washed and incubated with the secondary antibody at 1:10,000 dilution for 1 h. Finally, the blots were visualized using the Immobilon Western Chemiluminescent HRP Substrate (Millipore Cat# P36599) detection system.

### 2.8 Immunofluorescence

Cells were seeded on specific coverslips in 12‐well plates. The cell slides were fixed in 4% paraformaldehyde for 10 min. Permeabilization was performed with 0.1% PBST (phosphate‐buffered saline and 0.1% Triton) for 15 min. After blocking with 5% BSA for 1 h, the cell slides were incubated with primary antibody at recommended dilutions overnight in 4°C and stained with Alex Fluor 488‐conjugated second antibody (Jackson Cat# 111‐545‐144) for 1 h protected from light. DAPI was applied for 1 min as a nuclear marker. Images were acquired with an Eclipse TCS‐SP5 inverted confocal microscope (Leica).

### 2.9 Wound healing capacity test

HEK293 cells were seeded into the 2‐well culture insert (Ibidi Cat# 81176) pre‐coated by Matrigel. After cell attachment for approximately 24 h to form an optically confluent monolayer, the culture insert was removed to create the wound gap and the cells were then cultured with serum‐free medium. The wound gap closure was monitored by taking pictures with an Eclipse inverted microscope (Leica) at different time points.

### 2.10 Statistical analysis

GraphPad Prism was used to calculate and plot the mean ± SEM of measured quantities. Significances were assessed by two‐way analysis of variance (ANOVA)/Mann–Whitney *U* test (nonparametric)/Student's *t* test (parametric), *p*‐value < 0.05 was considered to indicate statistically significant differences. *p < 0.05, **p < 0.01, ***p < 0.001 and ****p < 0.0001.

## 3 RESULTS

### 3.1 SLC39A5 depletion impairs ECM synthesis

**FIGURE 1:**
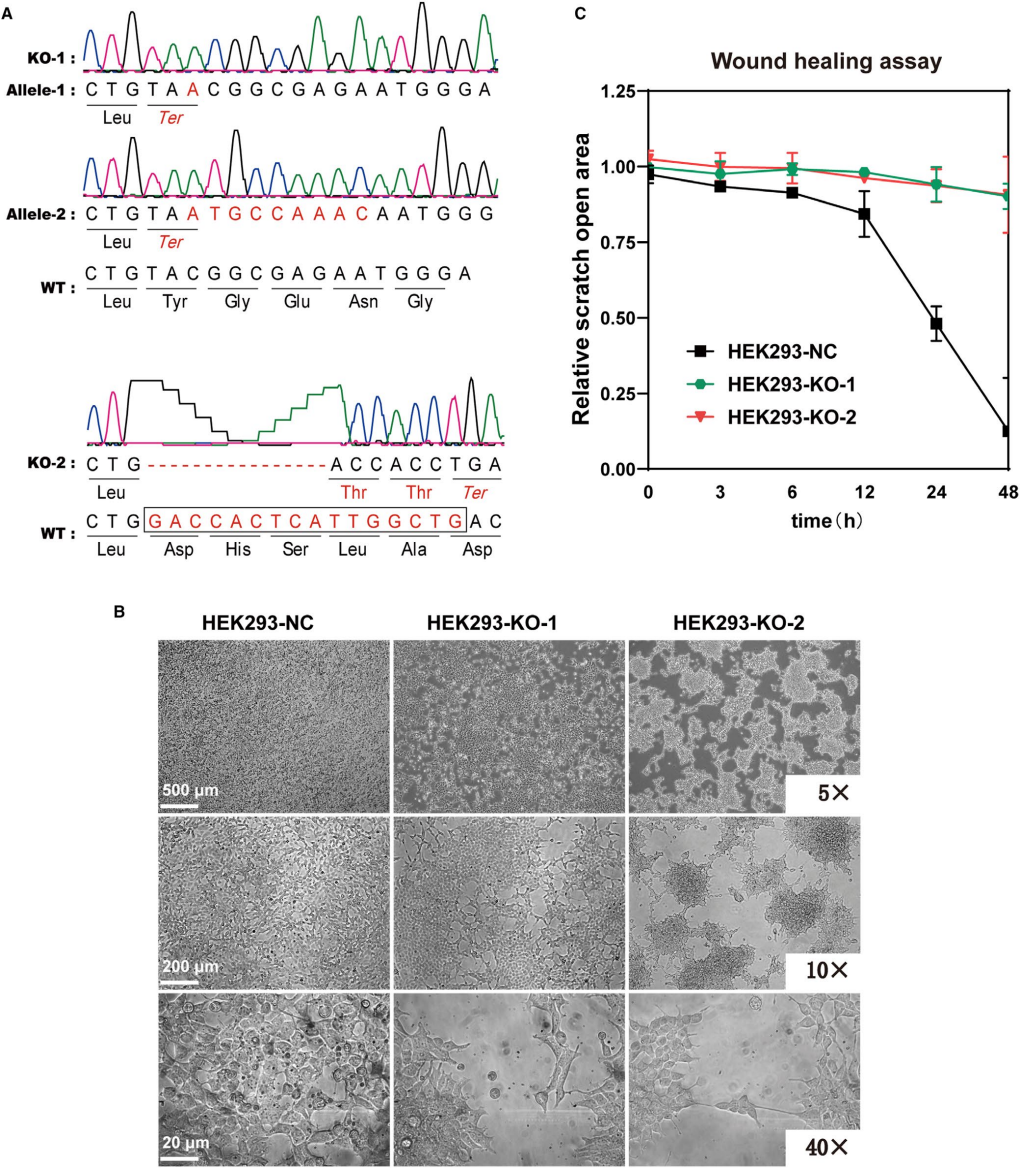
SLC39A5 depletion induces abnormal cell morphogenesis and migration. (A) shows the validation of the homozygous knockout (KO) HEK293 cell lines. Direct Sanger sequencing indicated the KO‐1 protein was early terminated when an A was inserted at position 141 (c.141insA, p.Y47*) or a 9‐bp insertion after a 7‐bp deletion from position 141 to 147 (c.141_147delinsATGCCAAAC, p.Y47*). KO‐2 harboured a 16‐bp deletion from position 439 to 454 (c.439_454del16, p.D147fs3*), inducing early termination after two missense threonine amino acids. (B) shows the typical morphology of the three SLC39A5‐related cell lines under 5×, 10× or 40× magnification, respectively. Two KO cell lines with substantial differences in the optically confluent monolayers were observed. (C) shows the relative scratch open area of the wound healing process of the three SLC39A5‐related cell lines. Fitting curves were obtained based on the scratch area images at different time points within 48 h. Compared with the control group, KO cells exhibited an evidently impaired wound healing ability

Two SLC39A5 knockout (KO) cell lines were developed using the CRISPR‐Cas9 system in the human embryonic kidney cell line (HEK293) (Figure 1A). Cell morphology anomalies were markedly observed in the SLC39A5‐depleted cells. After regular growth for 2 days, the KO cells revealed a tendency to form small cell aggregates instead of a typical optically confluent monolayer (Figure 1B). This phenotype suggested a potential impairment or abnormality of ECM generation. Considering the influence of ECM dynamics on cell migration, invasion and morphogenesis, we immediately investigated the cellular motility and migratory capacity of the SLC39A5‐depleted HEK293 cells. Scratch assay was performed in both groups. A 2‐well culture insert generated a uniform gap in the confluent monolayer, and wound healing was imaged at different time points. The results showed that the gap in the control group was completely healed after 24 h, while the wound gap in the KO group was not able to close (Figure S1; Figure 1C).

**FIGURE 2:**
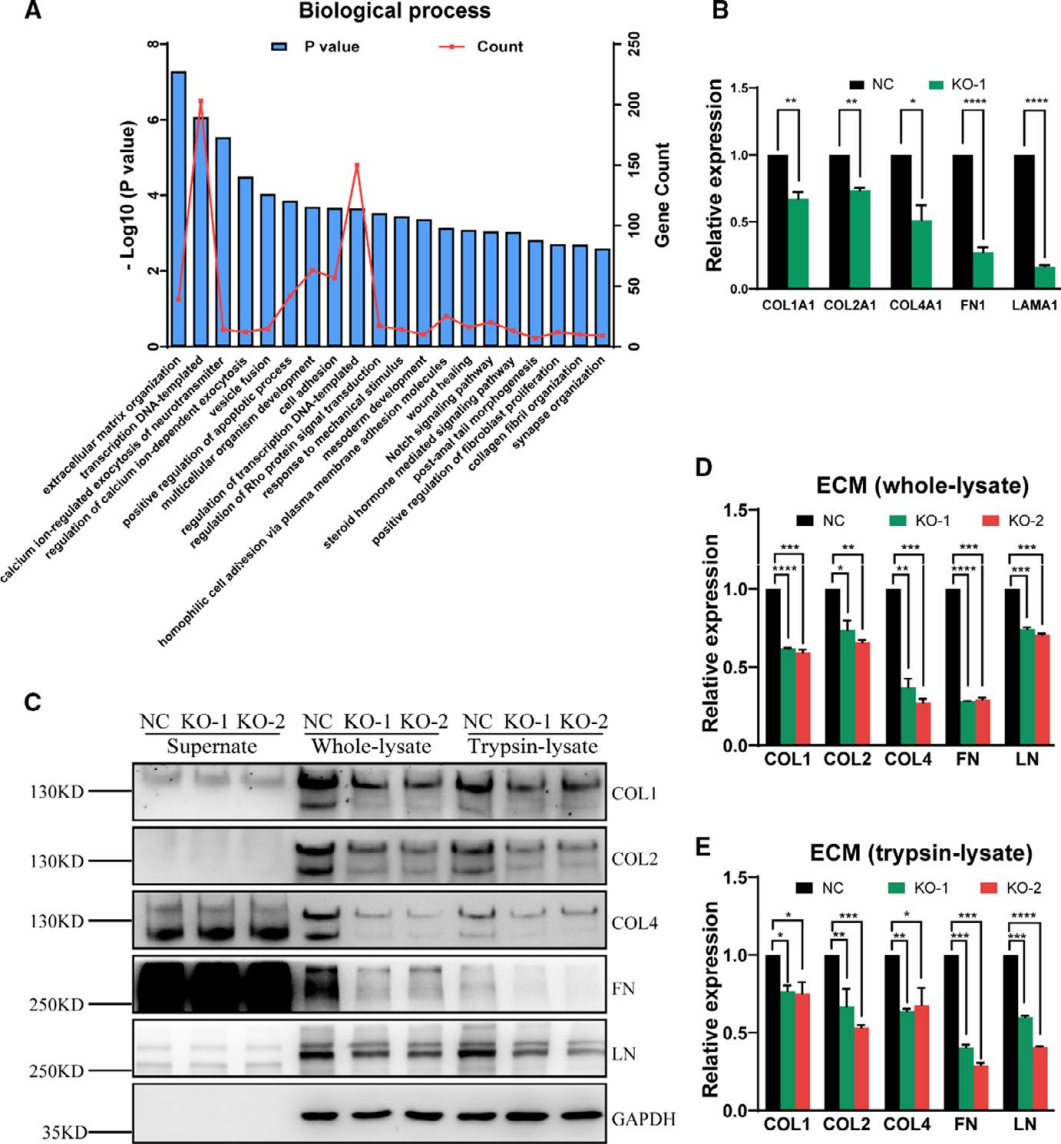
SLC39A5 depletion impairs extracellular matrix (ECM) synthesis. (A) shows the pathway enrichment analysis for the differentially expressed genes between the control and KO subpopulations of HEK293 cells. Pathway analysis was performed using the Database for Annotation Visualization and Integrated Discovery (DAVID) Bioinformatics Resources, with an enrichment *p*‐value cut‐off of 0.01. (B) shows the qRT‐PCR validation of the differentially expressed genes of the ECM members indicated by RNA‐seq. (C–E) shows the expression of several ECM components (COL1, COL2, COL4, FN and LN) in the supernatant, whole‐cell lysate or intracellular lysate (trypsin digested) of wild‐type (WT) and SLC39A5 knockout (KO) HEK293 cells. All tested ECM components were decreased in both the whole lysate and intracellular lysate. (B) and (C) show statistical analyses of the ECM components in the whole lysate and intracellular lysate, respectively. *p < 0.05, **p < 0.01, ***p < 0.001 and ****p < 0.0001

To investigate the overall alteration caused by SLC39A5 depletion, RNA‐sequencing (RNA‐seq) was performed using a SLC39A5 knockout cell line (KO‐1). In total, 810 upregulated and 1207 downregulated transcripts were predicted. All differentially expressed genes were subjected to Gene Ontology (GO) functional annotation analysis using the Database for Annotation Visualization and Integrated Discovery (DAVID) (https://david.ncifcrf.gov/). As shown in Figure 2, the significantly enriched genes and pathways were mainly associated with ECM organization, cell adhesion, wound healing and collagen fibril organization (Figure 2A). The qRT‐PCR results also validated the RNA‐seq predictions (Figure 2B), identifying transcriptional downregulation of certain ECM members under SLC39A5 depletion conditions. Later, immunoblot assay was used to assess the expression of Collagen I (COL1), Collagen Ⅱ (COL2), Collagen Ⅳ (COL4), fibronectin (FN) and laminin (LN) based on their robust expression in the ocular system. The results showed that contents of the candidate proteins in both whole‐cell lysate and intracellular lysate (trypsin digested) were significantly decreased (Figure 2C–E) (the bands of COL4/FN in the supernatant were found to be false positives (Figure S2)), suggesting that SLC39A5 depletion dysregulated the ECM constituents. Immunofluorescence also verified the decrease in these ECM proteins (Figures S3 and S4).

### 3.2 SLC39A5 depletion suppresses TGF‐β signalling

**FIGURE 3:**
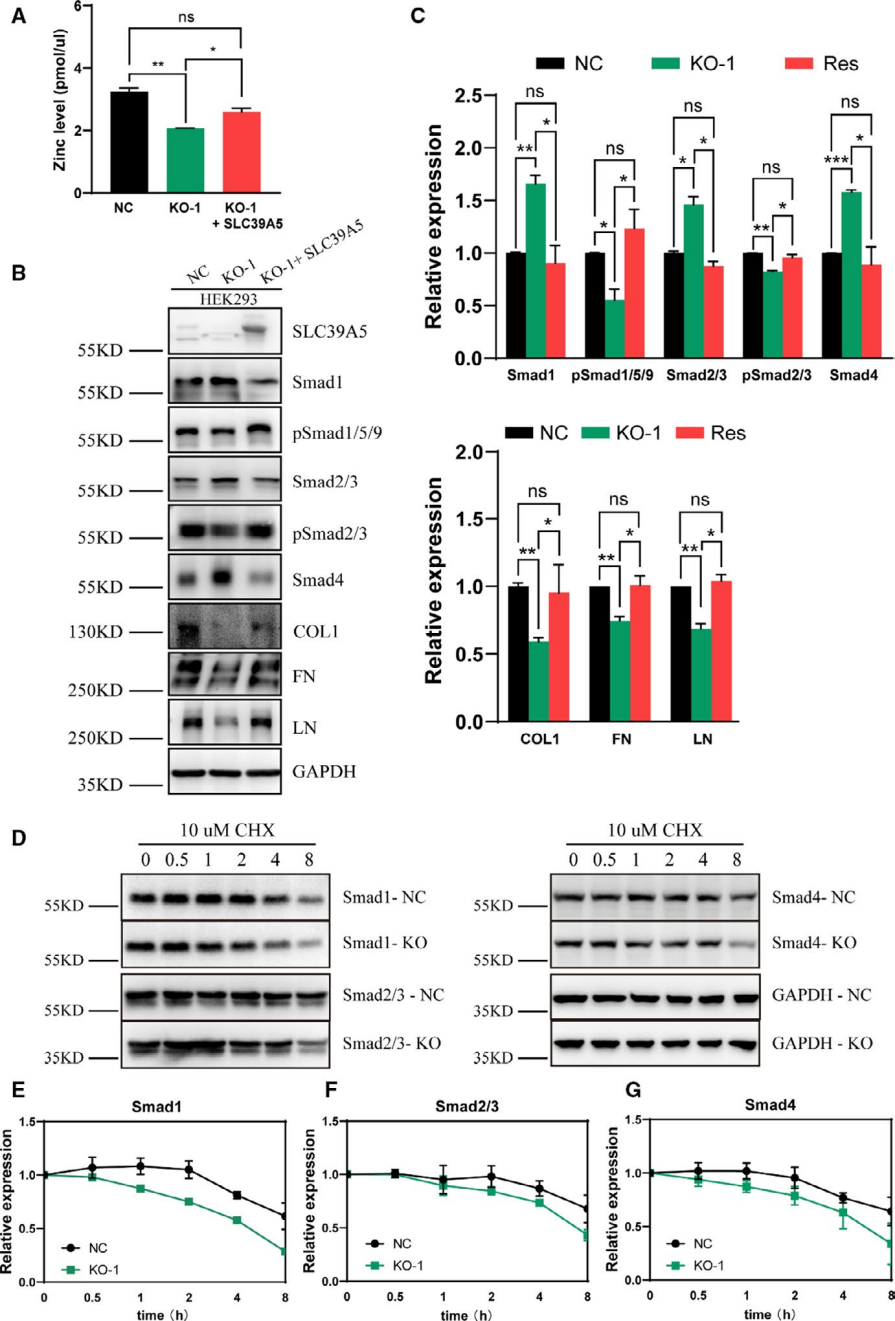
SLC39A5 depletion suppresses the TGF‐β signalling. (A) show the intracellular zinc level of the knockout (KO) cells was reverted after SLC39A5 re‐expression via lentivirus infection. (B,C) show the rescue of expression of the extracellular matrix members (COL1, FN and LN) and Smad proteins (Smad1, pSmad1/5/9, Smad2/3, pSmad2/3 and Smad4) in the knockout (KO) cells after SLC39A5 re‐expression via lentivirus infection. (D–G) show the degradation of cycloheximide (CHX)‐treated Smad1, Smad2/3 and Smad4 at different time points within 8 h. Degradation curves (E–G) were obtained based on statistical analyses of the Western blotting results (D). *p < 0.05, **p < 0.01 and ***p < 0.001

The transcription of collagens, FN and LN is closely related to TGF‐β signalling, and we have already determined that mutations of *SLC39A5* dysregulated TGF‐β signalling previously.^15^ Considering the transcriptional downregulation of collagens, FN and LN in SLC39A5 KO cells, we hypothesized that reduced SLC39A5 expression would inhibit the TGF‐β signalling pathway. Hence, we examined the expression of Smad proteins, which lie on the centre of the TGF‐β signalling pathway. The results showed an evident upregulation of total Receptor‐Smads (R‐Smads) (Smad1, Smad2/3) expression along with a significant decrease in phosphorylated Smad expression (pSmad1/5/9, pSmad2/3) (Figure S5). Additionally, Co‐Smad (Smad4) expression was also increased, while inhibitor‐Smad (Smad7) was not affected (Figure S5), suggesting a suppressed activation of the TGF‐β signalling, which was confirmed through immunofluorescence (Figures S6 and S7). The qRT‐PCR performed to assess the transcriptional levels of Smad1, Smad2 and Smad4 in KO cells showed increased mRNA expression of the tested proteins (Figure S8). To validate the phenotype of inhibited TGF‐β signalling activation and insufficient ECM synthesis resulting from SLC39A5 depletion, rescue experiments were carefully carried out. As shown in Figure  3, re‐expression of SLC39A5 restored intracellular zinc level (Figure 3A). TGF‐β signalling activation and ECM synthesis were also rescued (Figure 3B,C), wherein the total contents of R‐Smads and Smad4, the phosphorylation state of R‐Smads, and the expression of COL1, COL2, COL4, FN and LN were back to normal as expected.

The case of total increase and phosphorylation decrease in the content of Smad proteins prompted an examination of the stability. We used cycloheximide (CHX) to evaluate the half‐life of candidate Smads. The results showed that all Smad proteins tested revealed an accelerated degradation rate even with higher primary expression, suggesting a shorter half‐life of Smad proteins under SLC39A5‐depleted conditions (Figure 3D–G).

### 3.3 SLC39A5 depletion induces zinc deficiency and destabilizes Smad protein

**FIGURE 4:**
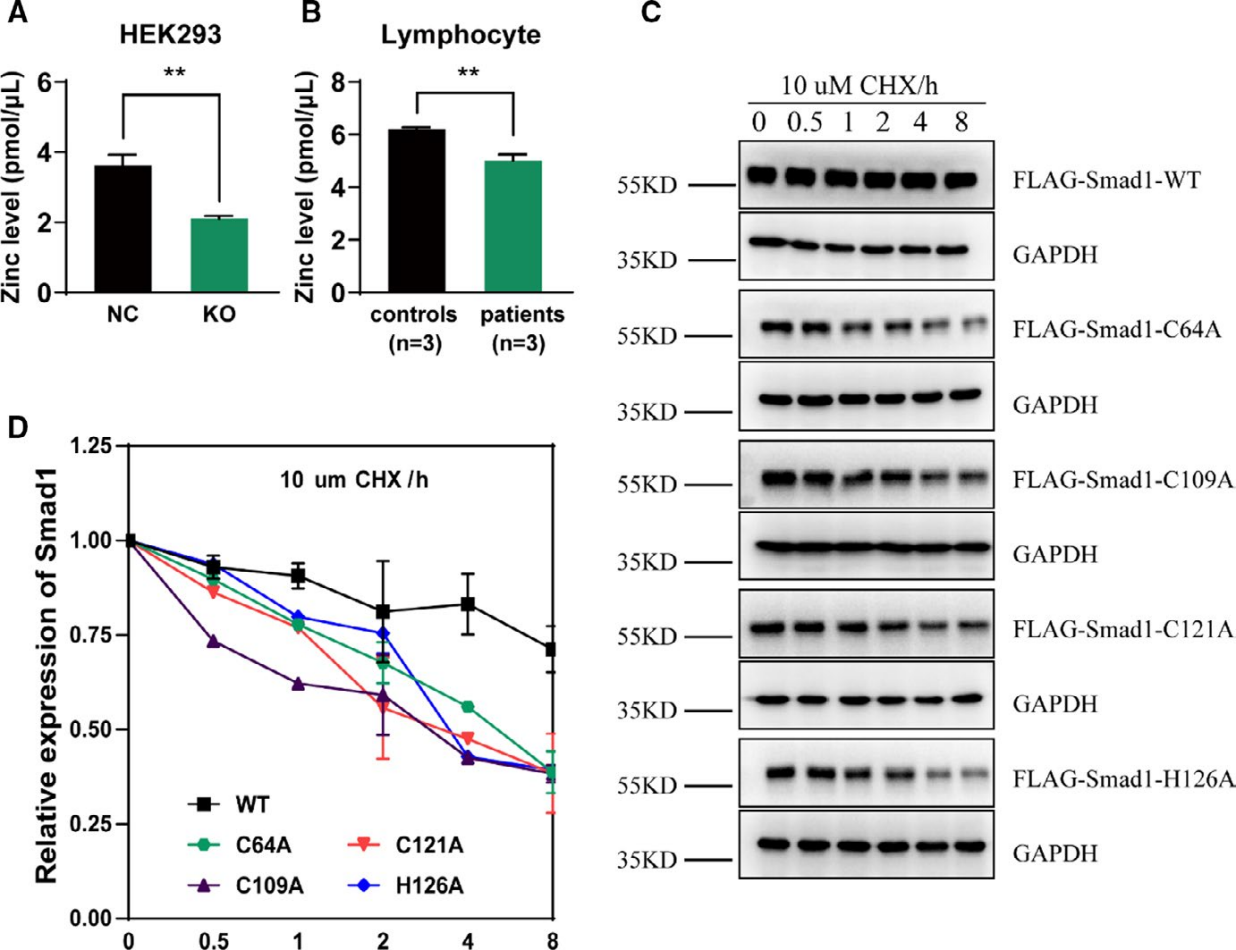
SLC39A5 depletion induces zinc deficiency and destabilizes Smad proteins. (A) shows zinc quantification of the SLC39A5‐related HEK293 cell lines. The average intracellular zinc level was lower in knockout (KO) cells than that in the control group. (B) shows zinc quantification of the lymphocyte cells from a high myopia pedigree carrying SLC39A5 mutations reported previously.^15^ The average intracellular zinc levels of the lymphocytes of patients (M16345, M16346 and M16349) were markedly lower than those of the normal controls (M16344, M19118 and M21932). (C,D) show the degradation of the cycloheximide (CHX)‐treated wild‐type or zinc‐binding site mutant Smad1 protein at different time points within 8 h of CHX treatment. Degradation curves (D) were created based on statistical analyses of the Western blotting results (C). **p < 0.01

The TGF‐β pathway is a zinc‐regulated pathway (Figure S9), while SLC39A5 is a zinc transporter.^17^, ^18^ These facts prompted us to confirm the involvement of zinc homeostasis in TGF‐β signalling alteration in SLC39A5 KO cells. Thus, zinc quantification was performed to detect intracellular zinc levels. As shown in Figure 4, SLC39A5 depletion significantly decreased the average intracellular zinc levels in the KO group (Figure 4A). We further evaluated the zinc level in lymphocyte cells generated from a high myopia pedigree, previously reported to harbour a SLC39A5 mutation^15^ and also observed lower average intracellular zinc levels in the patient cohort, similar to that observed in the KO cells (Figure 4B).

DNA‐binding capacity of R‐Smad requires zinc chelation,^19^ and the shorter half‐life of Smad proteins caused by SLC39A5 depletion indicated that zinc may also affect the Smad protein stability. To evaluate protein stability, four recombinant Smad1 plasmids, each carrying a single binding site mutant (C64A, C109A, C121A and H126A), were constructed and transferred to HEK293 cells, along with a wild‐type (WT) plasmid. As expected, the CHX assay showed a similar shorter half‐life of the mutant Smad1 proteins and a faster degradation rate (Figure 4C,D), confirming the hypothesis that zinc chelation is required for Smad protein stability. According to this hypothesis, the reestablishment of zinc homeostasis would be a novel strategy to rescue the phenotypes in SLC39A5 KO cells. Thus, we selected the most widely expressed zinc efflux protein SLC30A1 (20), as a target. Surprisingly, silencing SLC30A1 counterbalanced the influence of SLC39A5 depletion, which significantly reverted intracellular zinc level close to normal (Figure S10) and normalized the TGF‐β signalling pathway and ECM synthesis (Figure S11).

## 4 DISCUSSION

*SLC39A5* was first identified as a high myopia pathogenic gene in our previous study^15^ and was later confirmed as one of the top three genes contributing to nonsyndromic high myopia.^16^, ^20^, ^21^, ^22^, ^23^ However, the mechanisms by which SLC39A5 contributes to high myopia are not well understood. Here, we demonstrated that SLC39A5 may play a role in the pathogenesis of high myopia by modulating TGF‐β signalling–mediated ECM synthesis through the regulation of intracellular zinc homeostasis. SLC39A5 depletion significantly lowered the intracellular zinc level, which destabilized Smad proteins, and further inhibited TGF‐β signalling–mediated ECM synthesis, which ultimately contributed to the pathogenesis of high myopia.

Zinc homeostasis is crucial for all organisms as it serves as a catalytic or structural cofactor for multiple types of proteins. Zinc homeostasis depends on the transport of zinc in both directions across the plasma membrane and both in and out of various vesicular compartments.^24^ The SLC39 family facilitates zinc transport into the cytoplasm in the opposite direction to SLC30 family members.^25^ Within this network, SLC39A5 is a specific zinc transporter.^17^, ^18^, ^26^ Our work confirmed the zinc uptake characteristic of SLC39A5, in which SLC39A5 depletion caused an intracellular decline in zinc levels in HEK293 or lymphocyte cells. Once zinc homeostasis was disrupted, the catalytic or structural stability of proteins was impaired, suggesting that SLC39A5 depletion, indirectly, caused this impairment.

Zinc has a profound effect on stabilization of zinc‐binding proteins,^27^, ^28^, ^29^, ^30^, ^31^ since the proteins responsible for cellular zinc buffering will bind zinc transiently; therefore, their function will strongly depend on the cellular zinc status.^32^ It has been proposed that zinc chelation affects protein stability through various mechanisms. The human SOD1 protein requires zinc binding to facilitate the restructuring of apoproteins,^33^ while the putative protease of *Bacteroides thetaiotaomicron* (ppBat) requires zinc chelation to link the β‐strands to α‐helices in protein structures.^34^ In our study, the R‐Smads and Co‐Smad, which are pivotal to the BMP/TGF‐β signalling pathway,^35^ are zinc‐binding proteins. We found that Smad proteins were rapidly degraded during SLC39A5 deletion–induced zinc deficiency. Further results suggested that loose zinc coupling, facilitated by a mutant ion‐binding site, directly destabilized the exogenous Smad1 proteins, which clearly confirms the necessity of zinc chelation in maintaining the protein stability of Smads, while disturbances in zinc homeostasis induce protein instability.

Zinc binding has also been implicated in protein phosphorylation and subcellular translocation.^36^ Non‐zinc–bound protein kinase C delta (PKCδ) showed a significantly different structure near its phosphorylation site, compared with when it was zinc‐bound. Subcellular translocation of PKCδ was also influenced by intracellular zinc. These findings expand our understanding of the phosphorylation decrease in R‐Smads during SLC39A5‐depleted conditions. Since R‐Smads are directly phosphorylated by upstream receptor kinases, which then form heteromeric complexes with Smad4 to translocate into the nucleus,^37^ the zinc deficiency induced by SLC39A5 depletion may barely maintain the Smad protein in a favourable structure for phosphorylation, and may lead to nucleus translocation retention, further impairing the TGF‐β signalling.

It is well recognized that TGF‐β signalling directly affects ECM dynamics or triggers it when mediating other biological processes.^38^, ^39^, ^40^, ^41^ Herein, suppressed TGF‐β signalling in the zinc‐deficient conditions restrained ECM synthesis in HEK293. Recently, ECM synthesis and remodelling have attracted significant attention in high myopia aetiology studies.^8^, ^9^, ^10^, ^42^ We found that the transcription of all ECM proteins tested (COL1, COL2, COL4, FN and LN) was decreased under zinc‐deficient conditions. ECM‐induced cell morphogenesis and migration were also affected. Overall, these results indicate the presence of a zinc‐TGF‐β signalling‐ECM synthesis regulation flow. According to this hypothesis, the SLC39A5‐induced zinc deficiency suppresses TGF‐β signalling, leading to insufficient ECM synthesis, which may ultimately contribute to high myopia development. We further validated this hypothesis by performing rescue experiments. As expected, of SLC39A5 re‐expression, which recovers zinc influx, and SLC30A1 silencing, which blocks zinc efflux, could identically rescue the alteration of R‐Smad phosphorylation and ECM synthesis in SLC39A5‐depleted HEK293 cells, by re‐establishing zinc homeostasis.

Upon reviewing the relationship between zinc and high myopia, we found plenty of indications of zinc involvement in high myopia development.^43^ First, zinc is present abundantly in the ocular system, including the retina and choroid; thus, zinc deficiency produces a variety of ocular manifestations. Furthermore, a number of high myopia gene products are zinc‐related proteins. Among them, ZNF644, ZFHX1B, ZC3H11B and SCO2 are zinc finger proteins, and HGF and IGF1 are positively regulated by intracellular zinc concentration. Additionally, certain pathogenic genes (*LAMA1* and *TGFB1*) may even be regulated by intracellular zinc, according to the zinc‐TGF‐β signalling‐ECM synthesis flow. Finally, multiple small molecules that serve as chaperones for disease‐related genes or pathways are zinc‐related proteins. Smad proteins, together with their anchors (SARA and HGS), are either zinc‐binding or zinc finger proteins. These findings strongly highlight the correlation between zinc and high myopia.

In summary, our data suggest that depletion of SLC39A5 induces zinc deficiency, which restrains TGF‐β signalling–mediated ECM synthesis, thus possibly contributing to high myopia pathogenesis. Zinc homeostasis appears to be a dominant aetiological factor of high myopia development.

## CONFLICT OF INTEREST

The authors confirm that there are no conflicts of interest.

## AUTHOR CONTRIBUTION

**Shanshan Dong:** Conceptualization (equal); Data curation (lead); Formal analysis (lead); Investigation (lead); Methodology (lead); Validation (lead); Writing‐original draft (lead). **Qi Tian:** Data curation (supporting); Resources (supporting). **Tengfei Zhu:** Data curation (supporting); Resources (supporting). **Kangli Wang:** Formal analysis (supporting). **Ganting Lei:** Formal analysis (supporting). **Yanling Liu:** Formal analysis (supporting). **Haofeng Xiong:** Methodology (supporting). **Lu Shen:** Methodology (supporting). **Meng Wang:** Methodology (supporting). **Rongjuan Zhao:** Methodology (supporting). **Huidan Wu:** Methodology (supporting). **Bin Li:** Writing‐review & editing (supporting). **Qiumeng Zhang:** Methodology (supporting). **Yujun Yao:** Writing‐review & editing (supporting). **Hui Guo:** Funding acquisition (supporting); Writing‐review & editing (supporting). **Kun Xia:** Conceptualization (supporting); Funding acquisition (supporting); Writing‐review & editing (supporting). **Lu Xia:** Conceptualization (lead); Writing‐review & editing (lead). **Zhengmao Hu:** Conceptualization (lead); Funding acquisition (lead); Project administration (lead); Supervision (lead); Validation (lead); Writing‐review & editing (lead).

## Supporting information

Supplementary Material

## Data Availability

The data that support the findings of this study are available from the corresponding author upon reasonable request.
