# Supplementary material for: SLC39A5 dysfunction impairs extracellular matrix synthesis in high myopia pathogenesis
Source: J Cell Mol Med. 2021 Jul 24;25(17):8432–41. doi: 10.1111/jcmm.16803 (PMC8419198; doi:10.1111/jcmm.16803)
Supplement: Supplementary file 1 — Supplementary Material [file JCMM-25-8432-s001.pdf]

S1

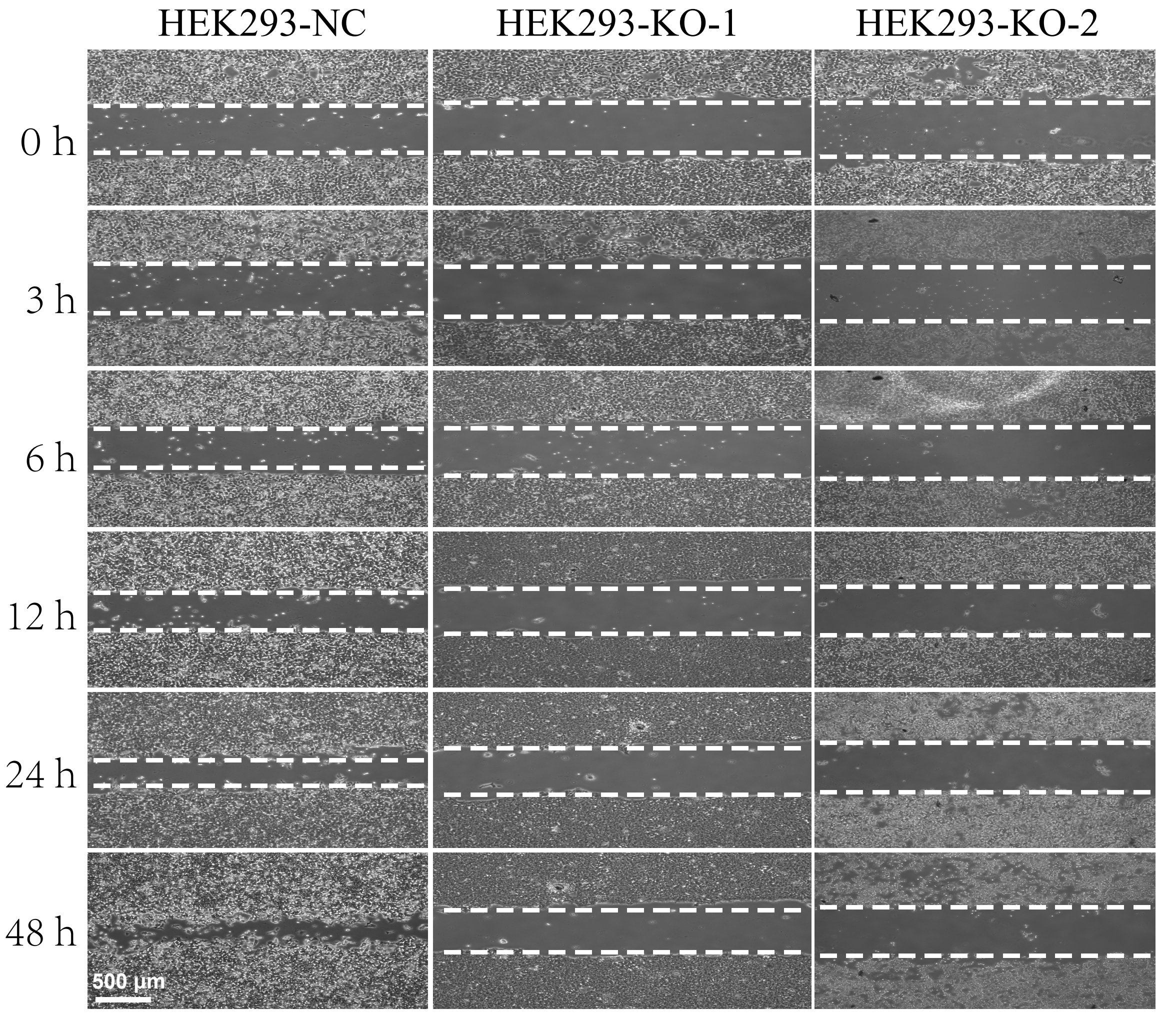

Figure S1: Scratch assay of the SLC39A5 related cell lines

S2

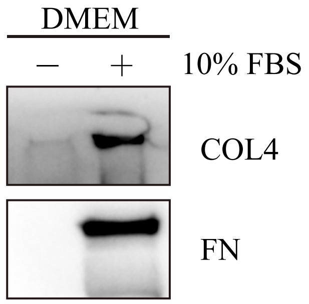

Figure S2: The COL4 / FN bands in supernatant were demonstrated false positives

S3

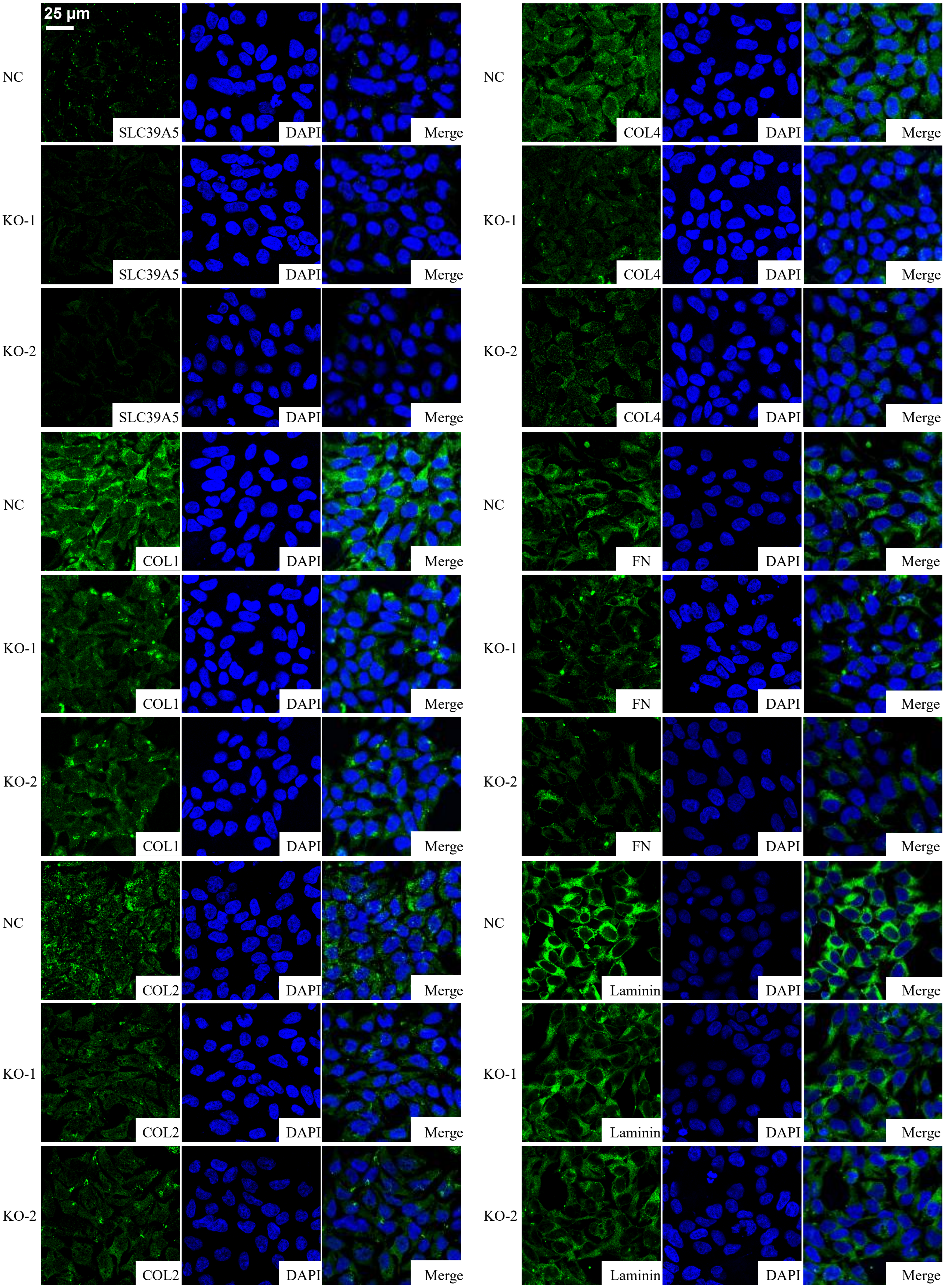

Figure S3: Immunofluorescence of ECM components in the SLC39A5 related cell lines

S4

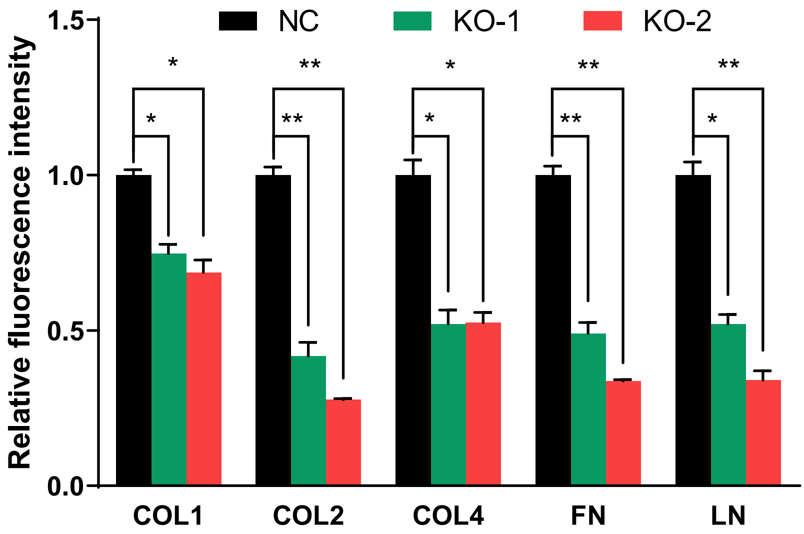

Figure S4: Statistical analysis of fluorescence intensity of ECM components in the SLC39A5 related cell lines

S5

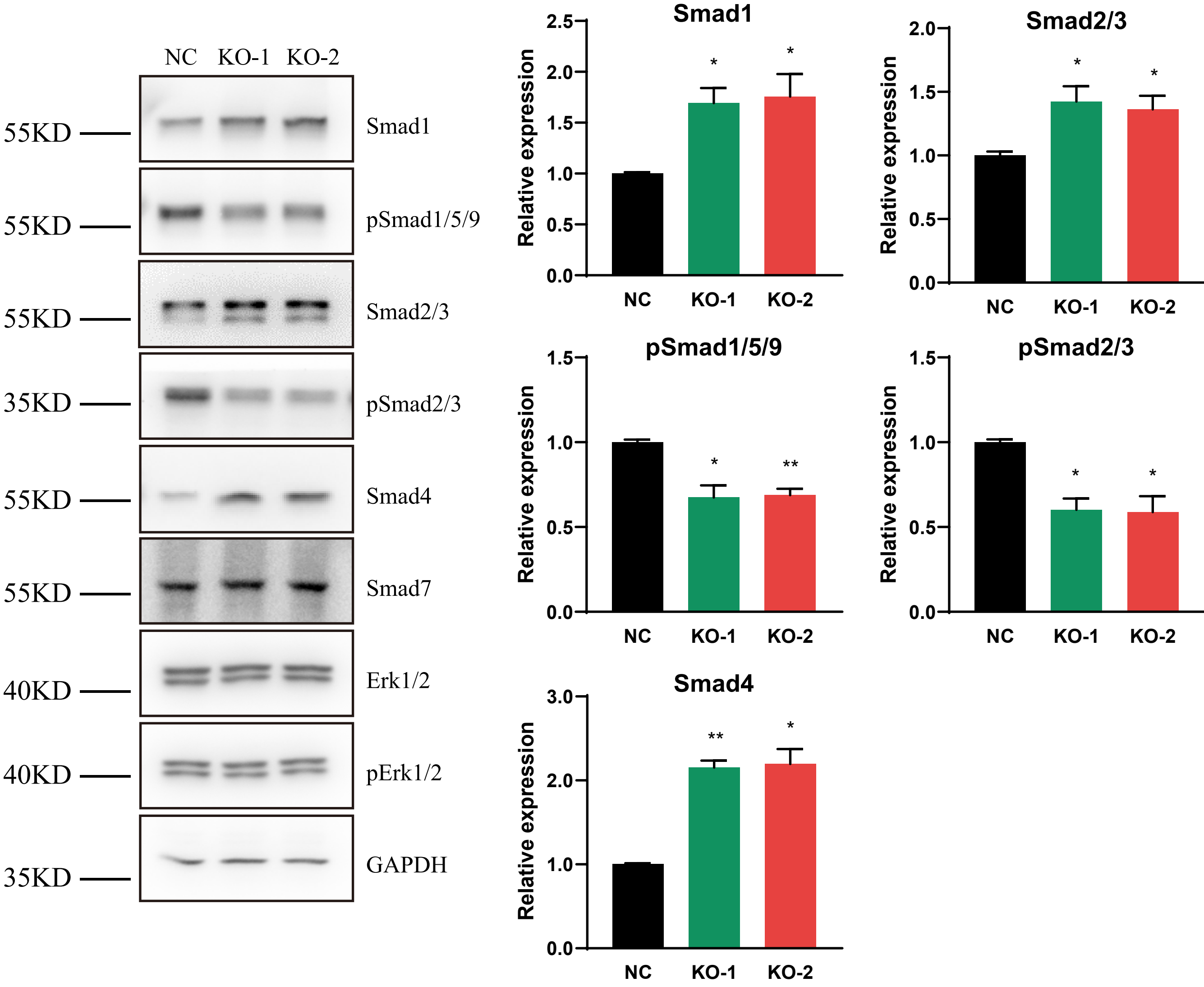

Figure S5: Western blot of Smad proteins in the SLC39A5 related cell lines

S6

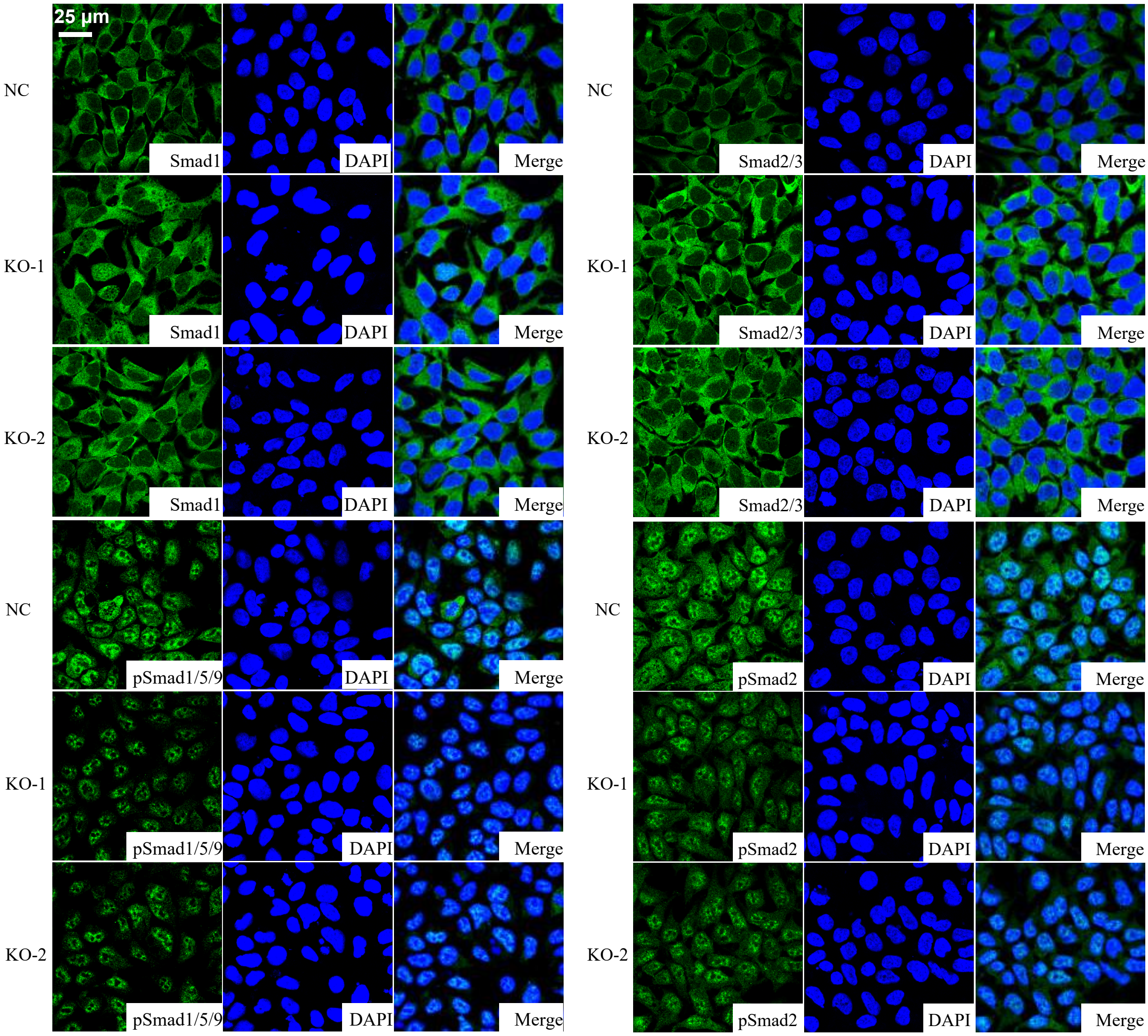

Figure S6: Immunofluorescence of Smad proteins in the SLC39A5 related cell lines

S7

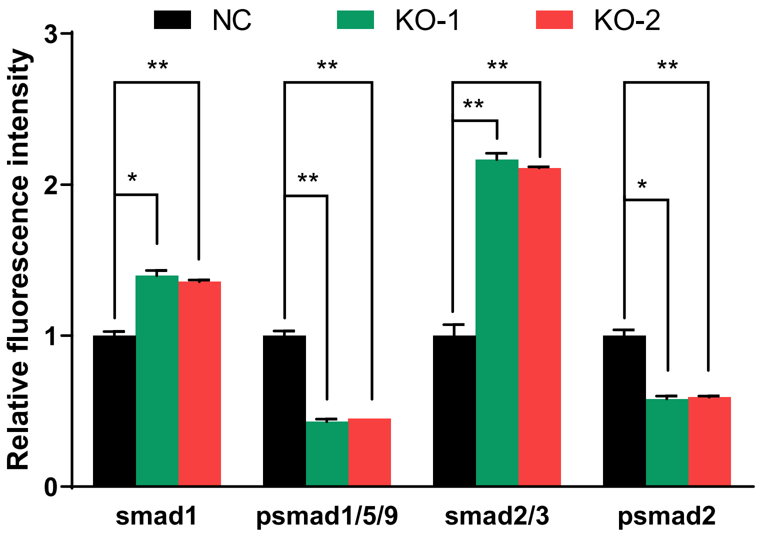

Figure S7: Statistical analysis of fluorescence intensity of Smad proteins in the SLC39A5 related cell lines

S8

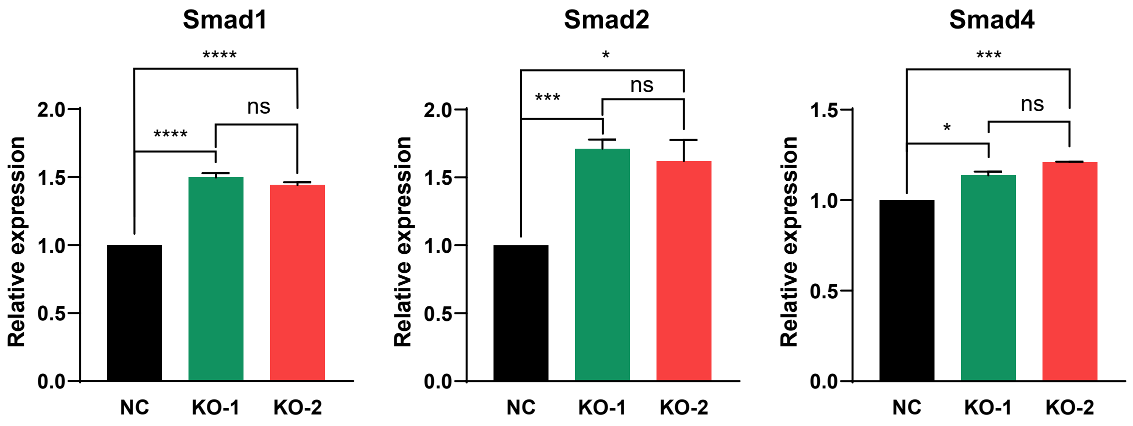

Figure S8: qRT-PCR of Smad1 / Smad2 / Smad4 in the SLC39A5 related cell lines

S9

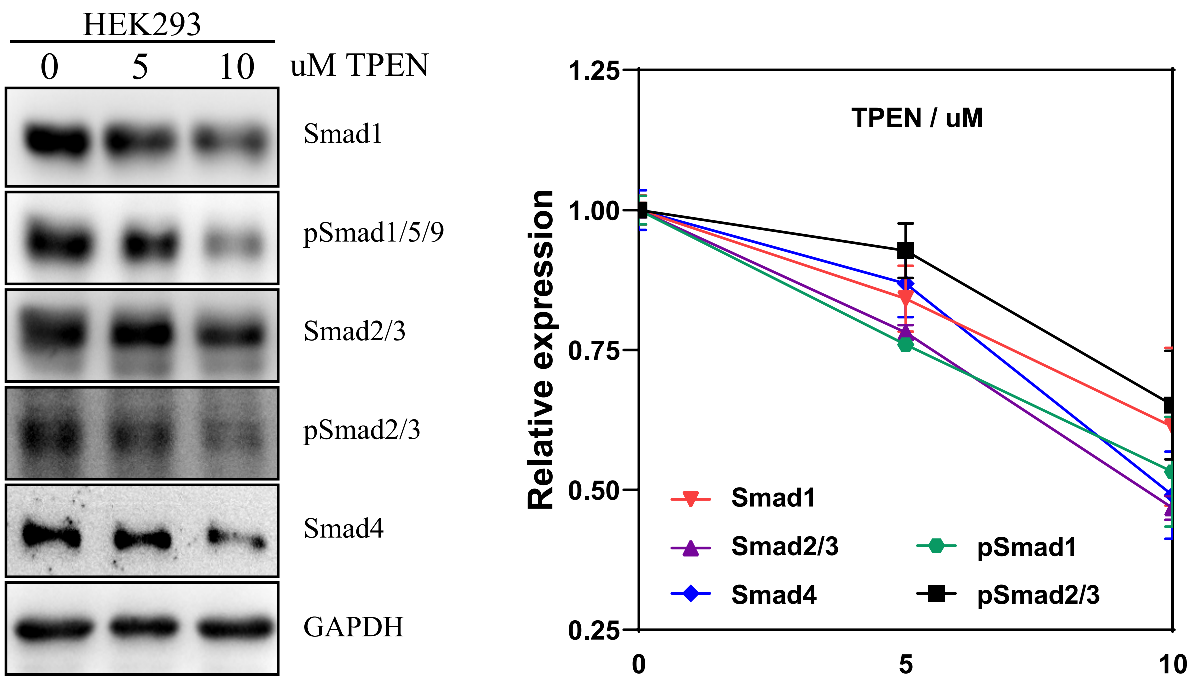

Figure S9: Western blot to investigate zinc's effect on TGF-β pathway

S10

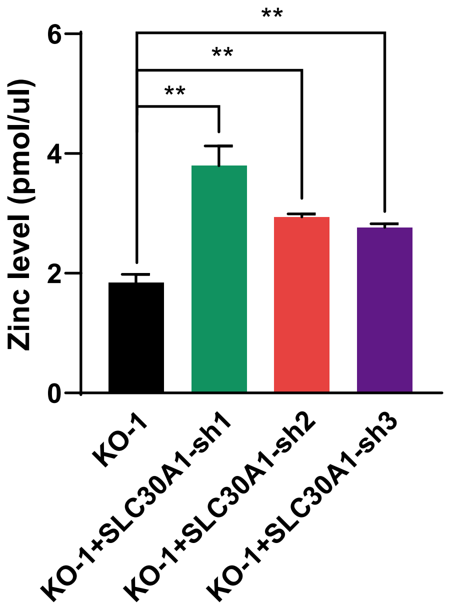

Figure S10: Zinc quantification assay on KO-1 cells silencing SLC30A1

S11

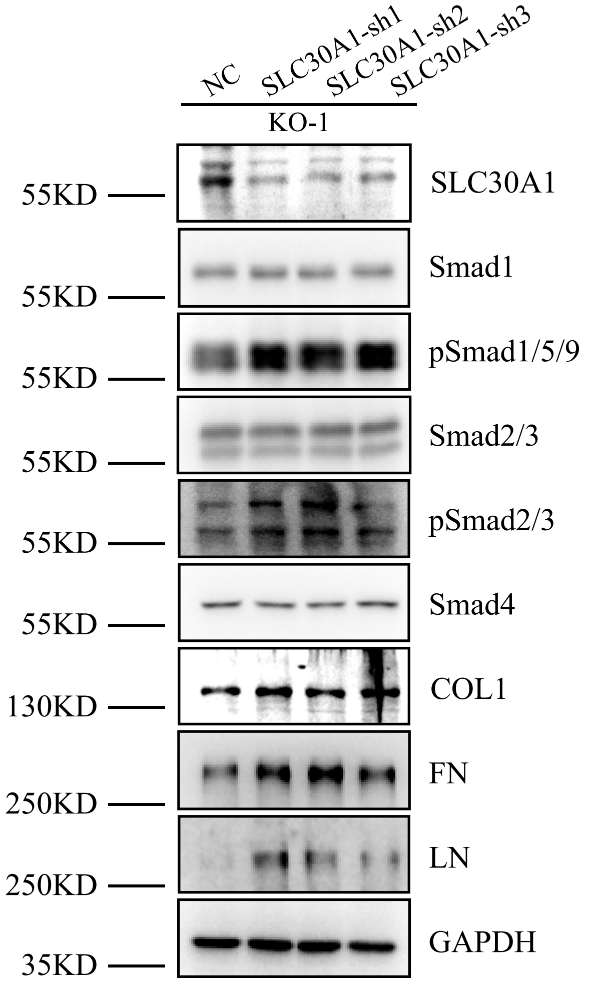

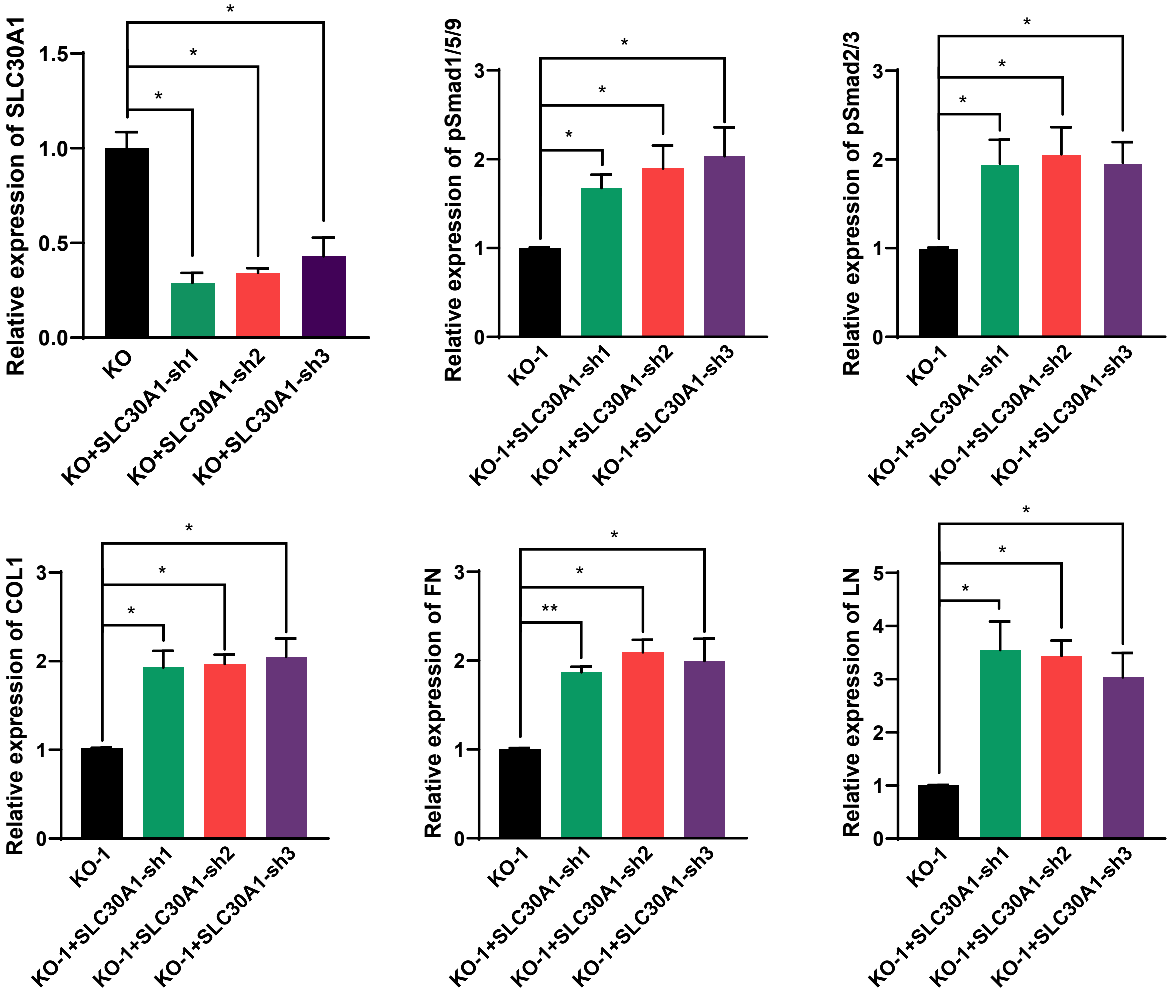

Figure S11: Western blot of ECM components and Smad proteins on KO-1 cells silencing SLC

Table S1. sgRNA-primers used for CRISPR-Cas9 knock out plasmid construction (5' to 3')

**Genes**	**Sequence**
SLC39A5-sg1-F	caccgGCGTCCCATTCTCGCCGTAC
SLC39A5-sg1-R	aaacGTACGGCGAGAATGGGACGCc
SLC39A5-sg2-F	caccgTCTGTTGCTGGACCACTCAT
SLC39A5-sg2-R	aaacATGAGTGGTCCAGCAACAGAc

Table S2. Primers used for CRISPR-Cas9 knock out validation (5' to 3')

**Genes**	**Sequence**
SLC39A5-sg1-validation-F	GGCAGTGTGAAGGGAGAAAG
SLC39A5-sg1-validation-R	CAAGGAGCTCGGTTCTGAAG
SLC39A5-sg2-validation-F	TCGTAGCCCTTTTGGTTGTC
SLC39A5-sg2-validation-R	CTAATCCCATGGCTCCTTCC

Table S3. Primers used for qRT-PCR (5' to 3')

**Genes**	**Sequence**
COL1A1-F	CGACAGAGGCATAAAGGGTC
COL1A1-R	GGAGACCGTTGAGTCCATCT
COL2A1-F	TCTACCCCAATCCAGCAAAC
COL2A1-R	GTTGGGAGCCAGATTGTCAT
COL3A1-F	TGCCATCCTGAACTCAAGAG
COL3A1-R	TGTCCACCAGTGTTTCCGT
COL4A1-F	GGGATGCTGTTGAAAGGTGAA
COL4A1-R	GGTGGTCCGGTAAATCCTGG
FN1-F	TGGAGATGAGTGGGAACGAA
FN1-R	CGGTCCCACTTCTCTCCAAT
LAMA1-F	TGGACTAGAGCTTGTGGACG
LAMA1-R	GGCGGTTTTGGGCTCATATG
Smad1-F	GCCCTGTACTTCCTCCTGTG
Smad1-R	TTGGGTTGCTGGAAAGAATC
Smad2-F	TCTTTGTGCAGAGCCCCAAT
Smad2-R	TGAGCCAGAAGAGCAGCAAA
Smad4-F	ATGTTTGGGTCAGGTGCCTT

Smad4-R	TGTCGATGACACTGACGCAA
SLC30A1-F	AAATTGGACCCCGCAGACCC
SLC30A1-R	GCCCTATCTTCTTCCAGTTCCAT
β-ACTIN-F	AATCTGGCACCACACCTTCTA
β-ACTIN-R	GATAGCAACGTACATGGCTGG
GAPDH-F	AGGGCTGCTTTTAACTCTGGT
GAPDH-R	CCCCACTTGATTTTGGAGGGA

Table S4. Primers used for Smad1 zinc binding site substitution construction (5’ to 3’)

**Genes/Variation**	**Sequence**
Smad1-C64A-F	GGGGAATGGTGACAGCGTTACTCGGTTGCC
Smad1-C64A-R	GGCAACCGAGTAACGCTGTCACCATTCCCC
Smad1-C109A-F	CAAAAGGAAACTCAGCGCATTCCAGTGGTT
Smad1-C109A-R	AACCACTGGAATGCGCTGAGTTTCCTTTTG
Smad1-C121A-F	GGTAGGGATTGATGGCGACCTCCTTCTGCT
Smad1-C121A-R	AGCAGAAGGAGGTCGCCATCAATCCCTACC
Smad1-H126A-F	CTACTCTCTTATAGGCGTAGGGATTGATGC
Smad1-H126A-R	GCATCAATCCCTACGCCTATAAGAGAGTAG

Table S5. Primers used for SLC39A5 rescue plasmid construction (5’ to 3’)

**Genes**	**Sequence**
SLC39A5-PAM1-Rescue-F	TTCTCGCCGTACAGTCCAAACAGCTGGGC
SLC39A5-PAM1-Rescue-R	GCCCAGCTGTTTGGACTGTACGGCGAGAA
SLC39A5-PAM2-Rescue-F	AGGTGGTCAGCTAATGAGTGGTCCAGCAACAGA
SLC39A5-PAM2-Rescue-R	TCTGTTGCTGGACCACTCATTAGCTGACCACCT

Table S6. Primers used for shRNA construction (5’ to 3’)

**shRNA**	**Target Sequence**
SLC30A1-shRNA-1	GGGCTGGACAACTTAACATGC
SLC30A1-shRNA-2	GGTCCTTGCTGGGTGCTATAT
SLC30A1-shRNA-3	GGAATCTGCTCTTATTCTTCT
Negative Control	CCAGATCAGGTGGCAATAAT
